# Dried Blood Spot PCR for Detection of Congenital Cytomegalovirus Infection and Disease

**DOI:** 10.1001/jamanetworkopen.2025.29837

**Published:** 2025-08-29

**Authors:** Mark R. Schleiss, Erin A. Osterholm, Ryan Shanley, Nelmary Hernandez-Alvarado, Emily Graupmann, Rebecca Kruc, Rick Jansen, Melissa Polonenko, Hannah Herd, Claudia Fernandez-Alarcon, Mark Blackstad, Sondra Rosendahl, Kirsten Coverstone, Mark McCann, Abbey Sidebottom, Whitney Wunderlich, Sasha Strul, Kathleen A. Kulus, Sheila C. Dollard, Tatiana M. Lanzieri

**Affiliations:** 1 Division of Pediatric Infectious Diseases and Immunology, Center for Infectious Diseases and Microbiology Translational Research, Minneapolis, Minnesota; 2 Division of Neonatology, Department of Pediatrics, University of Minnesota, Minneapolis; 3 Biostatistical Design and Analysis Center, Clinical and Translational Science Institute, University of Minnesota, Minneapolis; 4 MCC Biostatistics Core, Masonic Cancer Center, University of Minnesota, Minneapolis; 5 Department of Speech-Language-Hearing Sciences, University of Minnesota, Minneapolis; 6 Lions Children’s Hearing and ENT Clinic, M Health Fairview Masonic Children’s Hospital, Minneapolis, Minnesota; 7 Public Health Laboratory–Newborn Screening, Minnesota Department of Health, St Paul; 8 Care Delivery Research, Allina Health, Minneapolis, Minnesota; 9 Department of Ophthalmology, Minnesota Lions Children’s Eye Clinic, Minneapolis; 10 CentraCare/St Cloud Hospital, St Cloud, Minnesota; 11 Division of Viral Diseases, National Center for Immunization and Respiratory Diseases, Centers for Disease Control and Prevention, Atlanta, Georgia

## Abstract

This cohort study evaluates the performance of dried blood spot polymerase chain reaction (PCR) testing for the identification of congenital cytomegalovirus among infants in Minnesota.

## Introduction

Congenital cytomegalovirus (cCMV) infection is the leading infectious cause of disability among US children.^1^ Universal cCMV screening using dried blood spot (DBS) testing was implemented in Minnesota in 2023.^2^ Although DBS-based screening may miss some infants with cCMV infection, examining its sensitivity for identifying those with cCMV-related disease will be important to inform future newborn screening policymaking. We evaluated the sensitivity of DBS compared with saliva-based screening for detecting cCMV infection and cCMV-related disease.

## Methods

This prospective cohort study^3^ (eMethods in Supplement 1) enrolled newborns from February 2016 through December 2022 for cCMV screening by polymerase chain reaction (PCR) using saliva swabs and DBS at the University of Minnesota (UMN) laboratory and DBS alone at the US Centers for Disease Control and Prevention (CDC). The study was approved by the institutional review boards at participating institutions. Newborns were enrolled in the study with parental consent. We followed the STROBE reporting guideline. Screen-positive newborns with confirmed cCMV infection (ie, positive urine CMV PCR within 3 weeks of birth) were categorized as having cCMV disease if they had clinical signs at initial evaluation and/or findings compatible with disease as defined by international consensus criteria^4^ or if sensorineural hearing loss (SNHL) was diagnosed during follow-up.

We calculated the sensitivity for saliva PCR and DBS PCR for identification of infants with confirmed cCMV infection (analytical sensitivity) and cCMV disease (clinical sensitivity), respectively. We assessed prevalence of confirmed cCMV infection by study period, hospital nursery level, maternal demographic characteristics, and parity.^5^ We estimated prevalence and 95% CIs using log-binomial regression models and used the Wald χ^2^ test with 2-sided significance (*P* < .05) for all analyses, without adjusting for multiplicity.^5^ Analyses were performed in February 2025 using SAS, version 9.4 (SAS Institute Inc).

## Results

Among 23 641 screened newborns, 109 (0.5%) were CMV positive on 1 or more tests. On follow-up, 87 (79.8%) had confirmed cCMV infection, 19 (17.4%) were CMV negative, and 3 (2.8%) did not undergo urine testing. None were diagnosed with cCMV infection prior to screening. Final analytical sensitivity was 93.1% for saliva PCR; for DBS PCR, sensitivity was 72.4% and 79.3% by the UMN and CDC laboratories, respectively (Table 1).

**Table 1.** Performance of Saliva and DBS Polymerase Chain Reaction Testing for Identifying Confirmed cCMV Infection and Disease Among 23 638 Newborns, Minnesota, February 2016 to December 2022^a^

Test and outcome	Infants, No.		Performance (95% CI), %					
	Screened positive	Screened negative	Sensitivity	False negative	Specificity	PPV	False positive	NPV
**cCMV infection** ^b^								
Saliva screening								
Confirmed cCMV infection	81	6	93.1 (85.6-97.4)	6.9 (2.6-14.4)	99.9 (99.9-100.0)	83.5 (75.7-89.3)	16.5 (0.7-24.3)	100.0 (99.9-100.0)
No confirmed cCMV infection	16	23 535						
DBS at UMN								
Confirmed cCMV infection	63	24	72.4 (61.8-81.5)	27.6 (18.5-38.2)	100.0 (100.0-100.0)	98.4 (89.9-99.9)	1.6 (0.1-10.1)	99.9 (99.8-99.9)
No confirmed cCMV infection	1	23 550						
DBS at CDC								
Confirmed cCMV infection	69	18	79.3 (69.3-87.5)	20.7 (12.5-30.7)	100.0 (100.0-100.0)	97.2 (88.6-99.3)	2.8 (0.7-11.4)	99.9 (99.9-100.0)
No confirmed cCMV infection	2	23 549						
**cCMV disease^c^**								
Saliva screening								
Confirmed cCMV disease	20	1	95.2 (77.3-99.2)	4.8 (0.8-22.7)	99.7 (99.6-99.7)	20.6 (13.8-29.7)	79.4 (70.3-86.2)	100.0 (100.0-100.0)
No confirmed cCMV disease	77	23 540						
DBS at UMN								
Confirmed cCMV disease	17	4	81.0 (60.0-92.3)	19.0 (7.7-40.0)	99.8 (99.7-99.9)	26.6 (17.3-38.5)	73.4 (61.5-82.7)	100.0 (100.0-100.0)
No confirmed cCMV disease	47	23 570						
DBS at CDC								
Confirmed cCMV disease	19	2	90.5 (71.1-97.4)	9.5 (2.6-28.9)	99.8 (99.7-99.8)	26.8 (17.9-38.1)	73.2 (61.9-82.1)	100.0 (100.0-100.0)
No confirmed cCMV disease	52	23 565						

Abbreviations: cCMV, congenital cytomegalovirus; CDC, Centers for Disease Control and Prevention; DBS, dried blood spot; NPV, negative predictive value; PPV, positive predictive value; UMN, University of Minnesota.

^a^ A total of 3 infants missed follow-up urine testing within 3 weeks of age and therefore were excluded from the analysis; saliva testing results were positive for 2 and negative for 1, DBS testing results by the UMN laboratory were positive for 1 and negative for 2, and by the CDC, negative for 3. Among 87 newborns with confirmed cCMV infection, 55 (63%) were positive in all 3 screens, 13 (15%) in saliva only, 13 (15%) in saliva and 1 DBS screen, and 6 (7%) were negative in saliva and positive in at least 1 DBS screen.

^b^ cCMV infection was confirmed by urine polymerase chain reaction test in newborns with a positive screening result in either saliva swab or DBS by at least 1 laboratory. No confirmed cCMV infection indicates that cCMV infection was ruled out by urine polymerase chain reaction test in newborns with a positive screening result or newborns with negative screening results for saliva and DBS at both laboratories.

^c^ Confirmed cCMV disease, defined as clinical signs at initial evaluation or sensorineural hearing loss diagnosed during follow-up, identified in an infant with confirmed cCMV infection. No confirmed cCMV disease includes newborns with negative screening results for saliva and DBS in both laboratories, newborns with a positive screening result and a negative urine polymerase chain reaction test, and infants with confirmed cCMV infection who did not meet criteria for confirmed cCMV disease.

At the initial clinical evaluation, 15 of 87 newborns (17.2%) had clinical signs at birth (including 3 with congenital SNHL); 4 (4.6%) had isolated SNHL; and 68 (78.1%) had no clinical signs. Four infants—2 with and 2 without clinical signs at birth—were later diagnosed with delayed-onset SNHL at ages 4.0, 4.5, 6.0, and 60.0 months. Clinical information through age 3 years was available for all but 2 infants, who were lost to follow-up. Overall, 21 of 87 infants (24.1%) were classified as having cCMV disease (Table 2); the clinical sensitivity for cCMV disease was 95.2% (20 of 21 infants) for saliva, 81.0% (17 of 21 infants) for DBS (UMN), and 90.5% (19 of 21 infants) for DBS (CDC), corresponding to missed rates of 4.8%, 19.0% and 9.5%, respectively.

**Table 2.** Prevalence of Confirmed cCMV Infection by Selected Characteristics, Minnesota, February 2016 to December 2022^a^

Characteristic	Infants, No. with confirmed cCMV infection/No. screened	Prevalence (95% CI)	
		Confirmed cCMV infection per 1000	Ratio
Overall	87/23 638	3.7 (3.0-4.5)	NA
Study period			
February 2016 to March 2020	70/15 694	4.5 (3.5-5.6)	[Reference]
August 2020 to December 2021	6/4220	1.4 (0.6-3.2)	0.3 (0.1-0.7)
January 2022 to December 2022	11/3724	3.0 (1.6-5.3)	0.7 (0.4-1.2)
Hospital nursery^b^			
Postpartum unit	82/22 308	3.7 (3.0-4.6)	[Reference]
Neonatal intensive care	5/1107	4.5 (1.9-10.8)	1.2 (0.5-3.0)
Maternal characteristics			
Age group, y^c^			
≤24	14/2219	6.3 (3.7-10.6)	2.2 (1.1-4.6)
25-29	15/5241	2.9 (1.7-4.7)	[Reference]
30-34	37/9836	3.8 (2.7-5.2)	1.3 (0.7-2.4)
≥35	21/6338	3.3 (2.2-5.1)	1.2 (0.6-2.2)
Race			
Black	11/2417	4.6 (2.5-8.2)	[Reference]
White	70/16 929	4.1 (3.3-5.2)	0.9 (0.5-1.7)
Additional groups^d^	4/2127	1.9 (0.7-5.0)	0.4 (0.1-1.3)
Unknown	2/2165	0.9 (0.2-3.7)	NA
Hispanic ethnicity			
Hispanic or Latino	1/1931	0.5 (0.1-3.7)	0.1 (0.02-0.8)
Not Hispanic or Latino	84/17 267	4.9 (3.9-6.0)	[Reference]
Unknown	2/4440	0.5 (0.1-1.8)	NA
Parity^e^			
1	29/9970	2.9 (2.0-4.2)	[Reference]
2	45/7542	6.0 (4.5-8.0)	2.1 (1.3-3.3)
≥3	13/4576	2.8 (1.7-4.9)	1.0 (0.5-1.9)

Abbreviations: cCMV, congenital cytomegalovirus; NA, not applicable.

^a^ cCMV infection confirmed by urine polymerase chain reaction test in newborns with a positive screening result in either saliva swab or dried blood spot by at least one laboratory.

^b^ A total of 223 newborns were missing data for hospital nursery.

^c^ A total of 4 newborns were missing data for maternal age.

^d^ Additional groups include American Indian or Alaska Native, Asian, Native Hawaiian or Other Pacific Islander, and multiracial.

^e^ Parity includes the newborn enrolled in the study; 1550 missing parity.

The prevalence of confirmed cCMV infection was 3.7 per 1000 live births overall and varied by maternal age and parity. Prevalence declined substantially during the COVID-19 pandemic (Table 2).

## Discussion

Despite lower analytical sensitivity compared with saliva, DBS-based cCMV screening identified most cases of cCMV-related disease at birth or later in childhood. However, our DBS testing methods may not be easily reproducible in high-throughput, state-based screening programs. This requires continued evaluation in future studies.

This study has limitations. We assumed that infants with negative saliva and DBS tests were true negatives. Although initial diagnostic evaluations were coordinated by a single clinician, subsequent clinical follow-up occurred across multiple health systems throughout Minnesota, potentially leading to missed cases of cCMV infection and disease. Longitudinal long-term follow-up of this study population is warranted, and future studies will be important to identify predictors of cCMV-related disease.

## Notes

Supplement 1.
eMethods.

Supplement 2.
Data Sharing Statement
