# Supplementary material for: Dried Blood Spot PCR for Detection of Congenital Cytomegalovirus Infection and Disease
Source: JAMA Netw Open. 2025 Aug 29;8(8):e2529837. doi: 10.1001/jamanetworkopen.2025.29837 (PMC12397879; doi:10.1001/jamanetworkopen.2025.29837)
Supplement: Supplement 1. — eMethods. [file jamanetwopen-e2529837-s001.pdf]

Supplemental Online Content

Schleiss MR, Osterholm EA, Shanley R, et al. Dried blood spot PCR for detection of congenital cytomegalovirus infection and disease. *JAMA Netw Open.* 2025;8(9):e2529837. doi:10.1001/jamanetworkopen.2025.29837

eMethods

This supplemental material has been provided by the authors to give readers additional information about their work.

© 2025 Schleiss MR et al. *JAMA Network Open*.

eMethods

Study population/sites/study design

This prospective study was, as previously described[1], initially conducted at five Minneapolis/Saint Paul area newborn nurseries at the Fairview Health System (University of Minnesota [UMN] Masonic Children's Hospital, Fairview Ridges Hospital, Fairview Southdale Hospital), and Allina Health System (Abbott Northwestern Hospital and United Hospital). A sixth site, CentraCare/St. Cloud Hospital, was added in January 2021. Study enrollment initiated in February 2016, was halted from April to July 2020 due to the COVID-19 pandemic and resumed from August 2020 to December 2022[2]. The study was approved by the Institutional Review Boards at participating institutions.

For newborns enrolled in the study with parental consent, PCR on saliva swabs and DBS punches was performed as previously described[1]. Saliva swabs were tested by CMV DNA PCR at the UMN laboratory only, and DBS at both the UMN and the US Centers for Disease Control and Prevention (CDC). For DBS processed at the UMN, DNA was extracted from three 3-mm punches using a QIAcube HT extractor with the QIAamp 96 DNA kit (Qiagen) as previously described[1]. The material was then transferred to a deep-well 96 well plate (S-block; Qiagen), and automated extraction was performed using the QIAamp (Qiagen) 96 DNA version 1 protocol modified to elute DNA with 100 µL of water. Polymerase chain reaction testing of DBS DNA was performed in triplicate as previously described with 10 µl of eluate, using primers and probes corresponding to the HCMV *UL83* gene[3]. Dried blood spot DNA extraction at the CDC laboratory was a quick manual process using Quanta Extracta DBS buffer (QuantaBio), with eluate used directly for PCR. PCR was performed using PerfeCTa Fastmix II Low ROX master mix (QuantaBio) with TaqMan internal positive control to monitor for inhibition (ThermoFisher). The CMV Taqman probe MGBNFQ (ThermoFisher) and primers (Integrated

© 2025 Schleiss MR et al. *JAMA Network Open*.

DNA Technologies) targeted the CMV IE region.[4] The human gene ribonuclease P was amplified separately to monitor DNA extraction performance.

Any CMV-positive result for either sample prompted a recommendation from the Minnesota Department of Health Newborn Screening Program for follow-up urine testing within 3 weeks of birth, and consultation with an infectious disease specialist (M.R.S.). CMV PCR on urine specimens was performed at a Clinical Laboratory Improvement Amendments–certified diagnostic laboratory and used for definitive diagnosis of cCMV infection. For newborns with confirmed cCMV infection, follow-up diagnostic recommendations and clinical care were undertaken[5], and were generally based on an international cCMV consensus statement[6]. Neuroimaging using cranial ultrasound (cUS) examination was performed as part of the initial diagnostic evaluation[7]. Initial audiologic evaluation was undertaken by automated brainstem evoked response (ABR). Recommendations were made to have these infants followed by a pediatric audiologist once every three months. Referral to a pediatric ophthalmologist was routinely recommended to examine for any evidence of chorioretinitis. For this report, children with confirmed cCMV infection were categorized as having cCMV disease if they had at least one of the following: clinical signs at initial evaluation (i.e. petechiae, hepatomegaly, splenomegaly, microcephaly, or chorioretinitis); abnormal laboratory findings (i.e. thrombocytopenia, raised transaminases or direct bilirubin); abnormal cUS findings (i.e. ventriculomegaly, intracranial calcifications, periventricular echogenicity, or cortical or cerebellar malformations); or sensorineural hearing loss (SNHL). Congenital SNHL was diagnosed in an infant during the initial audiological evaluation after failing newborn hearing screening, and considered isolated SNHL if no other clinical signs were present during the neonatal period. Delayed-onset SNHL was diagnosed in an infant or child who passed the newborn hearing screening, had a normal initial audiological assessment, and SNHL detected during subsequent audiologic monitoring.

© 2025 Schleiss MR et al. *JAMA Network Open*.

Data collection

Data on maternal age, race/Hispanic ethnicity, and parity were collected through self-reported information from families, examination of medical records, interviews with the family, or medical history at the time of follow-up clinical evaluation. Maternal and newborn data, including laboratory results (complete blood count, platelet count, hepatic panel, urine and blood viral load) were entered into a REDCap database.

Sample size calculation

As previously described, we aimed to enroll 25,000 newborns in the screening study, assuming cCMV prevalence was 4.5 per 1,000 live births, which would allow us to identify 90 newborns with confirmed cCMV infection by saliva-based screening[1]. We hypothesized that DBS would identify most cases of cCMV disease; thus, DBS clinical sensitivity would approximate saliva clinical sensitivity, despite the DBS analytical sensitivity being lower than saliva's. We assumed the analytical sensitivity of DBS compared with saliva screening would be 60%, and the proportion with cCMV disease would be 17.5% among newborns detected by saliva screening and 37.5% among those detected by DBS screening. Thus, group sample sizes of 90 newborns with cCMV detected by saliva and 54 by DBS would achieve 80% power to detect a 20% difference between the group proportions of cCMV disease, with α of .05, using the 1-sided Mantel-Haenszel test. However, several unexpected developments during the study influenced final group sizes. The analytical sensitivity of DBS observed in the interim analysis was higher than projected, and study enrollment was halted for several months due to the COVID-19 pandemic, a time when cCMV prevalence decreased substantially[2]. Study enrollment ended in December 2022, with universal newborn screening for cCMV in the state of Minnesota commencing in February 2023. Therefore, we did not perform a statistical hypothesis test, and rather present the comparison of diagnostic performance for cCMV infection and cCMV disease.

Data analysis

© 2025 Schleiss MR et al. *JAMA Network Open*.

For assessing saliva and DBS test performance, we only included CMV-positive newborns by either saliva or DBS testing with a follow-up urine testing and assumed those with negative saliva and DBS tests in both laboratories were true negatives. We calculated final analytical sensitivity and specificity for saliva PCR and DBS PCR, as well as the proportions of true-positive (positive predictive value), false-positive, true-negative (negative predictive value), and false-negative results. Similarly, we assessed test performance for identification of infants with cCMV disease, that is the clinical sensitivity and specificity of saliva and DBS for identification of infants with cCMV disease among all newborns screened. The higher-than-expected analytical sensitivity of DBS screening observed in the study decreased the study power to find significant differences in clinical sensitivity.

We calculated prevalence of confirmed cCMV infection using the number of screened newborns as the denominator. Additionally, we compared prevalence of confirmed cCMV infection by study period, nursery type, and maternal demographics (age group, race, ethnicity) and parity. We estimated prevalence and 95% CIs using log-binomial regression models. We used the Wald χ^2 test with 2-sided significance ($P < 0.05$) for all analyses, without adjusting for multiplicity[2]. We present prevalence ratios (PRs) with 95% CIs. All analyses were performed with SAS, version 9.4 (SAS Institute Inc, Cary NC).

References

1. Dollard SC, Dreon M, Hernandez-Alvarado N, et al. Sensitivity of dried blood spot testing for detection of congenital cytomegalovirus infection. *JAMA Pediatr*. 2021;175(3):e205441. doi:10.1001/jamapediatrics.2020.5441.
2. Schleiss MR, Rosendahl S, McCann M, Dollard SC, Lanzieri TM. Assessment of congenital cytomegalovirus prevalence among newborns in Minnesota during the COVID-19 pandemic. *JAMA Netw Open*. 2022;5(9):e2230020. doi:10.1001/jamanetworkopen.2022.30020.

© 2025 Schleiss MR et al. *JAMA Network Open*.

3. Meyer L, Sharon B, Huang TC, et al. Analysis of archived newborn dried blood spots (DBS) identifies congenital cytomegalovirus as a major cause of unexplained pediatric sensorineural hearing loss. *Am J Otolaryngol*. 2017;38(5):565-570. doi: 10.1016/j.amjoto.2017.06.002.

4. Boppana SB, Ross SA, Shimamura M, et al. National Institute on Deafness and Other Communication Disorders CHIMES Study. Saliva polymerase-chain-reaction assay for cytomegalovirus screening in newborns. *N Engl J Med*. 2011;364(22):2111-2118. doi: 10.1056/NEJMoa1006561.

5. Rypka KJ, Schleiss MR. Impact of cytomegalovirus (CMV) on an academic pediatric infectious diseases outpatient clinic referral population, 2005-2020: Will the advent of universal congenital CMV (cCMV) screening change clinical practice referral patterns? *Int J Neonatal Screen*. 2024;10(1):14. doi: 10.3390/ijns10010014.

6. Rawlinson WD, Boppana SB, Fowler KB, et al. Congenital cytomegalovirus infection in pregnancy and the neonate: consensus recommendations for prevention, diagnosis, and therapy. *Lancet Infect Dis*. 2017;17(6):e177-e188. doi:10.1016/S1473-3099(17)30143-3.

7. Kruc RM, Osterholm EA, Holm T, et al. Cranial ultrasound findings in infants with congenital cytomegalovirus infection in a universal newborn screening study in Minnesota. *J Pediatric Infect Dis Soc*. 2024;13(8):413-420. doi: 10.1093/jpids/piae059.

© 2025 Schleiss MR et al. *JAMA Network Open*.
