# Supplementary material for: Dried Blood Spot PCR for Detection of Congenital Cytomegalovirus Infection and Disease
Source: JAMA Netw Open. 2025 Aug 29;8(8):e2529837. doi: 10.1001/jamanetworkopen.2025.29837 (PMC12397879; doi:10.1001/jamanetworkopen.2025.29837)
Supplement: Supplement 2. — Data Sharing Statement [file jamanetwopen-e2529837-s002.pdf]

Data Sharing Statement

Schleiss. Dried Blood Spot PCR for Detection of Congenital Cytomegalovirus Infection and Disease. *JAMA Netw Open*. Published September 02, 2025.
doi:10.1001/jamanetworkopen.2025.29837

Data
Data available: Yes
Data types: Deidentified participant data
How to access data: Contact Dr. Schleiss Email: schleiss@umn.edu
When available: With publication

Supporting Documents
Document types: Informed consent form, Other (please specify)
Additional Information: Protocol and data
How to access documents: Contact Dr. Schleiss schleiss@umn.edu Will be glad to share all data once IRB approval obtained
When available: With publication

Additional Information
Who can access the data: Data available after publication
Types of analyses: PCR methods; outcome data
Mechanisms of data availability: Conact Dr. Schleiss Email: schleiss@umn.edu
